# Effects of orthokeratology on the progression of low to moderate myopia in Chinese children

**DOI:** 10.1186/s12886-016-0302-5

**Published:** 2016-07-27

**Authors:** Mengmei He, Yaru Du, Qingyu Liu, Chengda Ren, Junling Liu, Qianyi Wang, Li Li, Jing Yu

**Affiliations:** 1 Department of Ophthalmology, Shanghai Tenth People’s Hospital, School of Medicine, Tongji University, No.301 Middle YanChang Road, ZhaBei District Shanghai, 200072 China; 2 Department of First Clinical Medical College, Nanjing Medical University, Nanjing, Jiangsu China

**Keywords:** Orthokeratology, Myopia control, Axial length, Effectiveness, Chinese

## Abstract

**Background:**

To investigate the effectiveness of orthokeratology (ortho-k) in reducing the development of myopia in Chinese children with low to moderate myopia.

**Methods:**

This was a retrospective study. In the ortho-k group, there were141 subjects, and the average age was (9.43 ± 1.10) years. The average spherical equivalent refractive error (SER) was (−2.74 ± 1.15)D, with examinations performed 1, 7, 30, and 90 days and 12 months after the patients started wearing ortho-k lenses. In the control group, there were 130 subjects, and the average age was (9.37 ± 1.00) years. The average SER was (−2.88 ± 1.39)D, with examinations performed every 6 months. Axial elongation, which is an important parameter reflecting the progression of myopia, was measured at baseline from the same IOLMaster each time by the same masked examiner and was compared between the groups after 1 year. The subjects were divided into two sub-groups according to age to further study the development of myopia at different ages. An unpaired *t*-test, paired *t*-test, Chi-square test and Spearman test were performed to analyze the data.

**Results:**

After 1 year, the average axial elongation was (0.27 ± 0.17) mm in the ortho-k lens group and (0.38 ± 0.13) mm in the control group, with a significant difference between the groups (*P* < 0.001). Axial elongation was not correlated with SER but had a negative correlation with initial age (ortho-k group: *r*_s_ = −0.309, *p* < 0.01; control group: *r*_s_ = −0.472, *p* < 0.01). The percentages of individuals with fast myopic progression (axial elongation > 0.36 mm per year) were 38.0 % among younger children (7.00 to 9.40 years) and 24.3 % among older children (9.40 to 12.00 years), whereas the respective percentages were 76.5 and 12.9 % in the control group. When SER ranged from -5.0D to −6.0D, the axial elongation in the ortho-k group was 57.1 % slower than that in the control group.

**Conclusions:**

Ortho-k lenses are effective in controlling myopic progression in Chinese children, particularly in younger children and in children with higher myopia.

## Background

Myopic progression in children is of great concern in Asian countries, such as China, Hong Kong, and India [1–3], and it has even become prevalent in Europe [4]. The incidence rate of refractive errors is the highest among the incidence rates of various eye diseases in the world [5]. Because of significant axial elongation of the eyeball, high myopia poses increased risks of retinal and vitreous detachments as well as other disorders, such as glaucoma and macular degeneration [6–8]. Thus, obtaining a better understanding of the mechanisms of myopia is important. Zhu et al. [9] concluded that the following induced abnormal axial growth of the eye: near accommodation lag, mechanical tension created by the crystalline lens or ciliary body, and peripheral retinal signal domination. Clinicians have been searching for treatments based on these controversial hypotheses, but no significant treatment result has been demonstrated yet.

Orthokeratology (ortho-k lenses), a special type of contact lens that uses rigid materials, ensuring the best oxygen transmission rate [10], has become increasingly popular for controlling myopic progression. A number of studies have demonstrated that the use of an overnight ortho-k lens is effective for controlling the progression of myopia [11–13]. Nonrandomized controlled trials (non-RCTs) have reported that axial length elongation in subjects wearing ortho-k lenses was 36 to 56 % slower than that in subjects who wore spectacles [12, 13]. The purpose of the present retrospective study was to evaluate both the effectiveness of the ortho-k lens in Chinese children with varying ages and varying degrees of myopia and the factors that influence this effectiveness.

## Methods

### Subjects

This was a retrospective study. The pertinent data from examination results were retrieved and rearranged from the medical records of children who came to our clinic for vision correction using spectacles or ortho-k lenses from 2013 to 2014. The children in the ortho-k lens group were selected from the patients who used ortho-k lenses for vision correction, and the children in the control group were selected from the patients who preferred single-vision spectacles to correct their myopia. They were all selected according to the inclusion criteria and were excluded if they conformed to one of the situations listed in the exclusion criteria (Table 1). Age, sex and spherical equivalent refractive error (SER) were also taken into consideration to minimize systematic bias when the children were enrolled. In total, there were 271 children (271 eyes) enrolled in this study. The ortho-k group included 141 children (62 were boys, and 79 were girls), and the children ranged in age from 7.00 to 11.28 (9.43 ± 1.10) years. The control group comprised 130 children (66 boys and 64 girls). The age of the children in the control group ranged from 7.10 to 11.40 (9.37 ± 1.00) years of age. For the baseline values, such as the male/female ratio (M/F ratio), SER, and axial length, no significant difference (*P* > 0.05, unpaired *t*-test and Chi-square test, Table 2) was observed between the ortho-k and the control groups. The children were divided into different myopic progression groups for analysis. The children with myopic progression less than the average annual growth (axial elongation ≤0.18 mm per year or ≤0.50D per year) were considered as slow progressors [14]. Children with axial elongation > 0.18 and ≤0.36 mm per year (myopic progression > 0.50D and <1.00D per year) were considered as moderate progressors [14]. Those with axial elongation > 0.36 mm per year (myopic progression > 1.00D per year) were considered as fast progressors [14]. The mean age of all children, or 9.4 years, was selected as the cut off value, and the children were divided into two age groups. The children who were 7.0 to 9.4 years of age were included in the younger group, and those who were 9.4 to 12.0 years of age were included in the older group. Both the ortho-k group and the control group were divided into two sub-groups based on basic SER: an SER between −0.50D and −3.00D was considered as low myopia, and an SER between −3.00D and −6.00D was considered as moderate myopia. In this study, only the data from the right eyes were analyzed, but during the treatment, the left eyes received the same treatment as the right eyes to ensure the best visual quality for the patients and to minimize the influence of the left eye on the axial length of the right eye.

**Table 1:** Inclusion and exclusion criteria

Inclusion criteria	Exclusion criteria
• 7.0 to 11.5 years old • SE: >−0.50D and < −6.00D in both eyes • With-the-rule astigmatism (axes 180 ± 30) < 1.50D • BCVA:(logMAR) of 0.10 or better in both eyes • Had an IOP of <21 mmHg • Maintained regularly scheduled visits and completed the 1-year follow-up	• Discontinued lens wear for a total of 30 days or less during the 1 year (criterion only for ortho-k group) • Previous experience withcontact lens wear or other treatment for myopia control • Strabismus at distance or near ornystagmus • Contraindication for ortho-k lens(e.g., limbus-to-limbus corneal cylinder and dislocated corneal apex) • Previous history of ocular surgery, trauma, or chronic ocular disease • Concurrent use of medications that may affect tear quality • Systemic or ocular conditions that may affect tear quality or contact lens wear (e.g., allergy and concurrent medication) or that may affect refractive development (e.g., Down syndrome, ptosis)

**Table 2:** Baseline data of subjects who completed the one-year follow-up examination in the ortho-k lens and control groups

	Ortho-k	Control	*P*-value
Total	*N* = 141	*N* = 130	
Age(y)	9.43 ± 1.10	9.37 ± 1.00	*P* = 0.643
M/F ratio	62/79	66/64	*P* = 0.362
SER(D)	−2.74 ± 1.15	−2.88 ± 1.39	*P* = 0.380
Axial length(mm)	24.71 ± 0.72	24.82 ± 0.77	*P* = 0.234

### Procedure

The ortho-k lenses (Ortho-K LK Lens; Lucid, Korea) were spherical 4-zone lenses that were made of gas-permeable lens material (Boston XO) with a nominal Dk of 100 × 10–11 (cm2.mLO2)/(sec. ml. mmHg). The nominal central thickness of the lenses was 0.22 mm (central lens thickness: 0.24 mm), and the diameter was 10.6 mm. All of the subjects in the ortho-k group were fitted with the lenses by a certified ophthalmic technician based on the manufacturer’s instructions. Subsequently, the lenses were dispensed to the patients. The patients were advised to wear the lenses for at least seven consecutive hours every night, and a cleaning procedure was necessary every time that they removed the lenses. In the control group, the patients wore single-vision glasses made of plastic lens material. These spectacles were prescribed by the same technician who fitted the subjects in the ortho-k lens group and were modified according to visual acuity (VA) and refractive changes during the follow-up period. Myopic progression was estimated based on changes in axial length in both groups, which were evaluated using a noncontact optic biometric device (IOLMaster (IOL: intraocular lens); Carl Zeiss Meditec AG, Jena, Germany). The axial length measurements were taken from the same IOLMaster each time by the same masked examiner.

Before the subjects in the two groups started wearing the lenses, their axial length, best-corrected VA(BCVA), VA, intraocular pressure (IOP), noncycloplegic manifest refraction, cycloplegic manifest refraction, corneal topography(Oculus, Wetzlar, Germany),corneal curvature, and corneal endothelial cells were assessed. The axial length was used to estimate the myopic progression, and the other examinations were just used to evaluate the health status of the patients before they started wearing the lenses and during their follow-up visit. Subjects in the ortho-k group were examined 1, 7, 30, and 90 days and 6 and 12 months after they started wearing the ortho-k lenses. The examinations were performed 3 h after removing the lenses and included measurements of the subjects’ VA, refraction, IOP, axial length, corneal integrity, and corneal topography and fluorescein staining of the corneal epithelium. Whenever any abnormal symptoms, such asphotophobia, redness, eye pain, or tearing occurred, wearing of the ortho-k lenses was stopped for an average of 1 month. If the symptoms were severe, the subjects received treatment. The subjects could not restart wearing the lenses until they were completely recovered because lens wearing may make keratitis more severe. In the control group, the examinations were performed every 6 months and included measurements of the subjects’ VA, IOP, and axial length. The ortho-k lenses and the spectacles were refitted according to VA and refractive changes to ensure the best visual quality for the patients.

### Data analysis

The data analysis was performed using SPSS software ver. 17.0 (SPSS Inc., Chicago, IL, USA). All data were distributed normally (Kolmogorov-Smirnov test, *P* > 0.05). The data are presented as the mean ± standard deviation. The difference in the M/F ratio between the groups was assessed using the Chi-square test. Unpaired t-tests were used to compare other baseline data between the groups to determine myopic progression over 1 year. Additionally, a paired *t*-test with Bonferroni correction was performed to compare the difference in axial length between the two groups, and an unpaired *t*-test was used to compare the difference in axial elongation between the groups. Factors that may affect axial elongation were examined using Spearman analysis. *P* < 0.05 was considered to be statistically significant.

## Results

The axial lengths in the ortho-k and spectacle groups at baseline are shown in Table 2. In the ortho-k group, the initial axial length was 24.71 ± 0.72 mm, and after 1 year, the axial length was 24.98 ± 0.70 mm. In the control group, the axial length increased from 24.82 ± 0.70 mm to 25.20 ± 0.78 mm. The axial elongation was 0.27 ± 0.17 mm and 0.38 ± 0.13 mm in the ortho-k and control groups, respectively. There was significant axial elongation at the first-year follow-up in both the ortho-k and the control groups (*P* < 0.001, paired *t*-test, Table 3). Thus, the axial increase in the ortho-k group was significant, with a 28.9 % smaller increase than that in the control group (*P* < 0.001, unpaired *t*-test).

**Table 3:** Axial length in the ortho-k and spectacle groups during the study period

	Ortho-k	Control
Before wearing the lens(mm)	24.71 ± 0.72	24.82 ± 0.77
After 1 year(mm)	24.98 ± 0.70	25.20 ± 0.78
Axial elongation(mm)	0.27 ± 0.17	0.38 ± 0.13
*P*-value	*P* < 0.01	*P* < 0.01

**Fig. 1:**
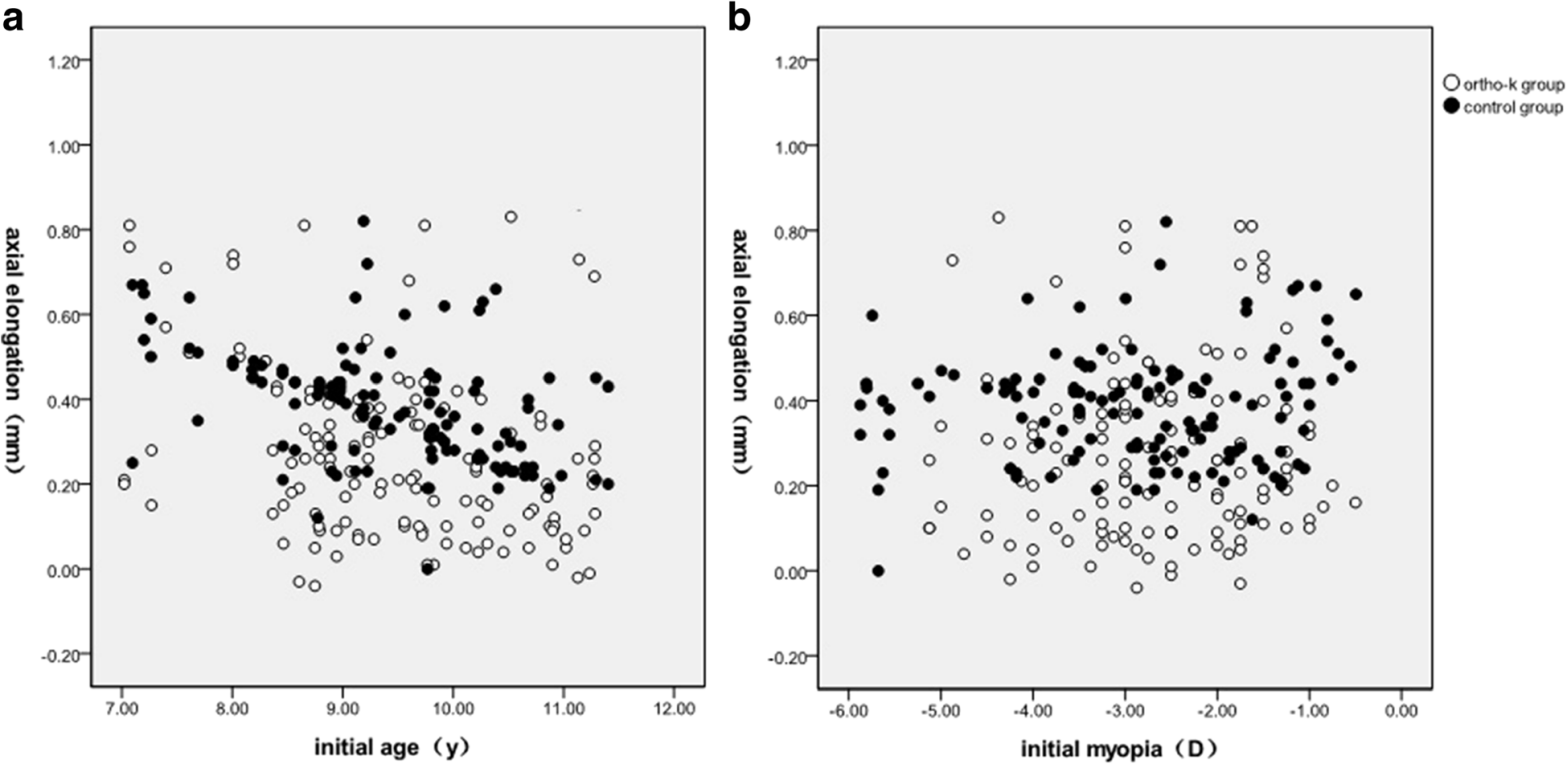
Scatter plots showing correlations of axial elongation with initial age (**a**) and SER at baseline (**b**) in the ortho-k and control groups

A Spearman test of axial elongation and initial age was performed for each group. In this study, there was a significant negative correlation between axial elongation and initial age in both groups during the 1-year period (ortho-k group: *r*_s_ = −0.309, *p* < 0.01; control group: *r*_s_ = −0.472, *p* < 0.01; Fig. 1a), which shows that younger myopic children will benefit more from ortho-k treatment than older myopic children. A Spearman test of axial elongation and SER was also performed. For the 1-year period, the increase in axial length was plotted against SER at baseline for both groups; however, there was no correlation between the changes in the axial length and initial myopia in either group of subjects (ortho-k group: *r*_s_ = 0.040, *p* = 0.638; control group: *r*_s_ = 0.140, *p* = 0.098, Fig. 1b).

**Fig. 2:**
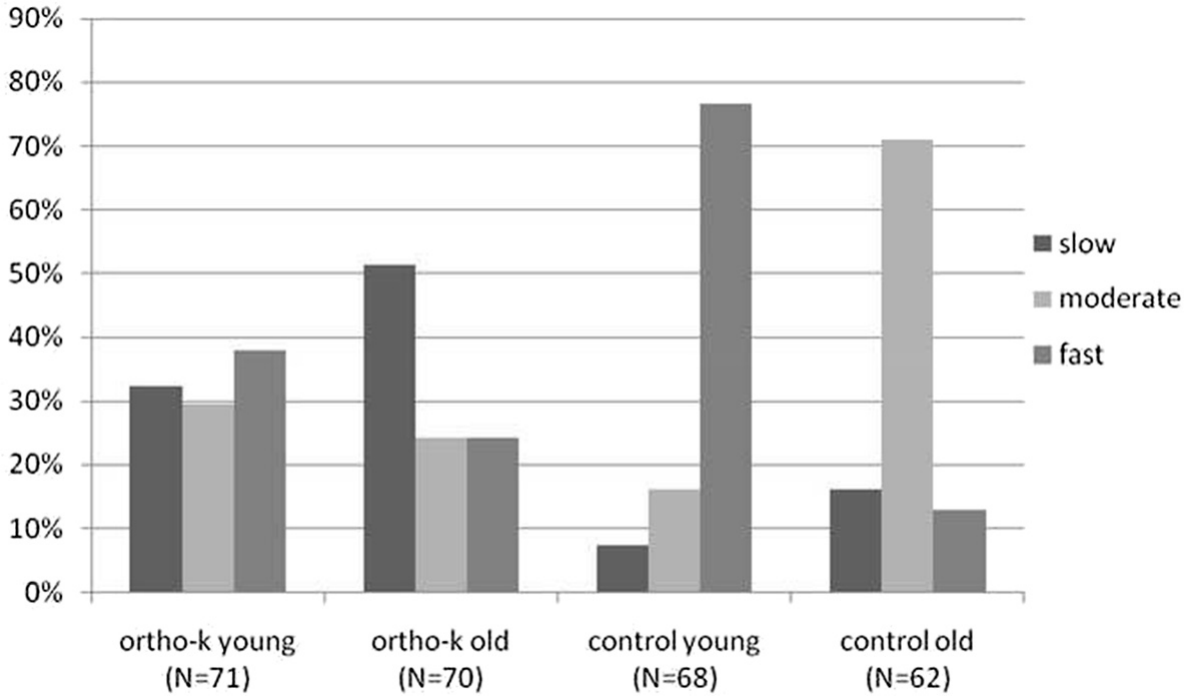
Percentage of subjects with different myopic progression speeds (slow, moderate, fast) in different age groups (younger, older) in the ortho-k and control groups

Comparing the ortho-k and control groups, 31.2 % of the ortho-k group consisted of fast progressors, which was a lower percentage than in the control group (46.2 %, Chi-square test, *P* < 0.001). The percentage of slow progressors decreased from 41.8 % in the ortho-k group to 11.5 % in the control group. To further compare myopic progression between younger and older children, we determined that the percentages of younger subjects with fast myopic progression were 38.0 and 76.5 % in the ortho-k and control groups, respectively, whereas the percentages among the older subjects were lower, reaching 24.3 and 12.9 % in the ortho-k and control groups, respectively. During the 1-year period, in the control group, the percentage of younger subjects with slow progression was only 7.3 %, while in the ortho-k group, it was 32.4 % (Fig. 2).

**Fig. 3:**
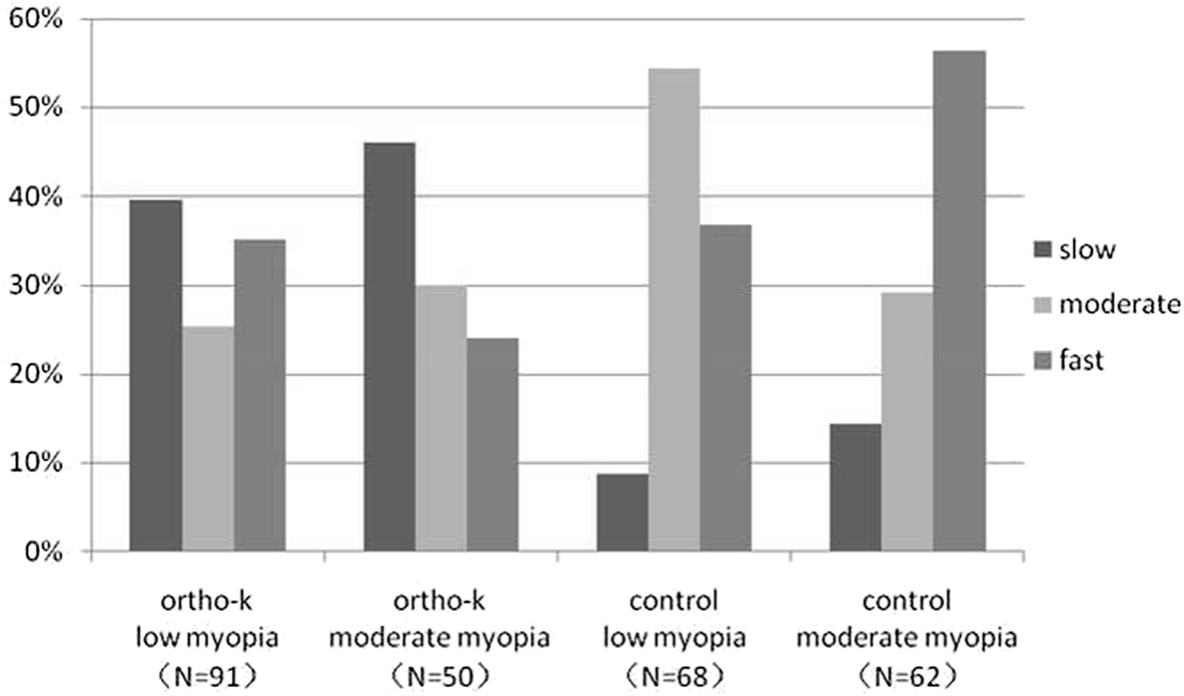
Percentage of subjects with different myopic progression speeds (slow, moderate, fast) among subjects withdifferent degrees of initial myopia in the ortho-k and control groups

**Fig. 4:**
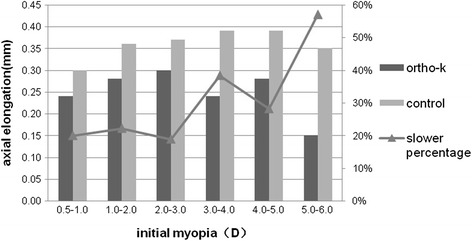
Axial elongation of subjects with different degrees of baseline SER in the ortho-k and control groups, with a slower percentage for the ortho-k lens group compared with the control group

Comparing the two groups, among those with moderate myopia, the proportions of low to moderate progressors did not show a significant difference (Fig. 3). Among the subjects with low myopia, the average axial elongation was 0.28 ± 0.18 mm and 0.38 ± 0.15 mm in the ortho-k and control groups, respectively, and this difference was statistically significant (*P* < 0.05). The same results were observed among the subjects with moderate myopia (*P* < 0.05) (Table 4). When comparing the subjects with low myopia and the subjects with moderate myopia, the elongation of axial length was 26.3 and 34.2 % slower in the ortho-k and control groups, respectively (Table 4). When the increases in axial length in the ortho-k and control groups were compared (Fig. 4), the ortho-k group had a notably shorter axial length. The axial elongation in the ortho-k group was slower than that in the control group, and the percentage showed a general trend of escalation. When SER ranged from −5.0D to −6.0D, the axial elongation in the ortho-k group (*N* = 11) was 57.1 % slower than that in the control group (*N* = 13) and was the slowest among the 6 sub-groups (Fig. 4).

**Table 4:** Axial length in different degrees of myopia inthe ortho-k and spectacle groups during the study period

	Total	Axial length (mm, initial)	Axial length (mm,1 year)	Axial elongation (mm)	*P*-value
Low myopia					
Ortho-k	*N* = 91	24.57 ± 0.72	24.85 ± 0.66	0.28 ± 0.18	<0.05
Control	*N* = 68	24.46 ± 0.61	24.85 ± 0.63	0.38 ± 0.15	<0.05
Moderate myopia					
Ortho-k	*N* = 50	24.97 ± 0.66	25.22 ± 0.71	0.25 ± 0.16	<0.05
Control	*N* = 62	25.34 ± 0.68	25.72 ± 0.69	0.38 ± 0.12	<0.05

In the current study, 18 of 141 subjects in the ortho-k group had previously had orthokeratology-associated infectious disease. Among them, most children experienced only slight discomfort, and only 3childrenhad a severe condition and received eye drop treatment before they restarted wearing the lenses.

## Discussion

The mechanisms of ortho-k lens-induced myopia reduction include central corneal flattening and thinning of the central corneal epithelium [15, 16] as well as thickening of the mid-peripheral cornea and peripheral vision myopic shift [17]. Studies advocate the use of reverse-geometry rigid contact lenses that are worn overnight to maintain the modified shape of the cornea and that are removed upon waking. Regular wear maintains the reshaping effect, thus temporarily controlling the myopia [18]. The data from the present study support the theory that the ortho-k lens can effectively control myopia by inhibiting axial elongation compared with traditional single-vision spectacles in Chinese children with low to moderate myopia. As shown in Table 5, different studies reporting the effectiveness of the ortho-k lens and other treatments were compared [9, 19–22]. Though these studies varied in their patient demographics, such as initial age, race/ethnicity, study period, and the method of treatment in the control group, the conclusions were nearly identical, reporting that the ortho-k lens was more successful in controlling myopia compared with other treatments, including physical and medical treatments.

**Table 5:** Studies on myopia control using ortho-k lens

Study (year)	Swarbrick HAet al. (2015) [19]	Meng-Jun Zhu et al. (2014) [9]	Jaime Paunéet al. (2015) [20]	Hiraortho-k a T et al. (2012) [21]	Lin HJ et al. (2014) [22]
Age (y)	10.8–17.0 years	7–14 years	9–16 years	8–12 years	7–17years
Race/ethnicity	East Asian	Chinese	Caucasian	Japanese	Chinese
Duration of study	1 year	2 years	2 years	5 years	3 years
With control group	yes	yes	yes	yes	yes
Control treatment	rigid gas-permeable (RGP)	spectacles refractive gradient (SRRG)	soft radial	spectacles	0.125 % atropine
Method of assignment	randomized	historic data	nonrandomized	randomized	self-selection
Initial SER	−2.43 ± 0.98	−4.29 ± 2.04	−0.75 ± 0.25	−1.89 ± 0.82	−4.25 ± 1.5
(Ortho-k, D)					
Initial SER (Control, D)	−2.39 ± 0.93	−4.24 ± 2.38	−0.76 ± 0.27	−1.83 ± 1.06	−4.0 ± 1.75
Increase in axial length in 1 y (Ortho-k, mm)	no change	0.16 ± 0.17	0.15 ± 0.10	0.99 ± 0.47	0.28 ± 0.08
Increase in axial length in 1 y (Control, mm)	0.09 ± 0.09	0.39 ± 0.21	0.26 ± 0.15	1.41 ± 0.68	0.38 ± 0.09
Reduction in myopia progression in 1 year	100 %	59 %	42.3 %	30 %	26.3 %

Many studies have shown that the ortho-k lens is effective in myopia control, but the mechanism by which the ortho-k lens might control myopia is still under debate. It was believed that the imaging properties of the fovea were the main reason for emmetropia in the past, but animal studies have indicated that compared with the fovea, peripheral visual signals play a more important role in emmetropia [23]. Peripheral hyperopia may act as a signal for eye growth [24]. Norton et al. proposed that relative peripheral hyperopic defocus in myopes may trigger axial elongation [25]. Thus, correction of peripheral hyperopia may be the mechanism by which the ortho-k lens suppresses myopic eye growth.

It was found that the axial length is related to the initial age at baseline [26], and studies have shown that myopic progression slows with age [27]. In the current study, a negative correlation was found between axial elongation and initial age (Fig. 1a). The percentage of fast progressors in the ortho-k group was 38.0 % among younger subjects and 24.3 % among older subjects, while the percentage of fast progressors in the control group was 76.5 % among younger subjects and 12.3 % among older subjects. Among younger children, the percentage of fast progressors was 38.0 % in the ortho-k group and 76.5 % in the control group, and among older children, the percentage of fast progressors was 24.3 % in the ortho-k group and 12.3 % in the control group (Fig. 2); these findings showed that the ortho-k lens had a better effect on controlling myopia compared with single-vision spectacles and that the younger group had a faster progression of myopia than did the older group. Donovan et al. [28] showed a similar result. Comparing the percentage composition within different age groups, the ortho-k group included 32.4 % younger subjects with slow progression and 38 % with fast progression, whereas the control group included 7.3 % with slow progression and 76.5 % with fast progression. Similar to the younger population, the ortho-k group included 51.4 % older subjects with slow progression and 24.3 % with fast progression, whereas the control group included 16.1 % with slow progression and 12.9 % with fast progression (Fig. 2). This study demonstrates that younger myopic children will experience a greater benefit from ortho-k treatment than will older myopic children. Hiraoka et al. [21] suggested that the earlier ortho-k lens treatment is initiated, the greater its inhibitory effect will be on axial growth.

Although in this study, there was no significant correlation between initial myopia and axial elongation (Fig. 1b), Fan et al. [26] noted that the higher the degree of myopia is, the faster the axial length will grow. As shown in our study, the percentage of fast progressors accounted for 56.4 % of moderate myopia in the control group (Fig. 3), which was comparable to Fan’s results. Previous studies have also reported that subjects with higher initial myopia benefitted the most from ortho-k treatment [12, 18]. Compared with the children with low myopia, children in this study who had an axial length indicating moderate myopia benefitted more from ortho-k treatment. Although there was no direct correlation between axial elongation and SER, this study generally showed that the treatment with the ortho-k lens led to slower axial elongation (Fig. 4). This study demonstrates that individuals with higher myopia would have better results with ortho-k lens treatment.

In addition, certain studies have reported that the ortho-k lens was a good treatment for myopia control, whereas other reports have demonstrated its adverse effects, such as variability in VA and sight-threatening infective keratitis and conjunctivitis [29–31]. Reviewing all reported cases of ortho-k-related keratitis between 2001 and 2007, the peak occurrence was in the early years of this 7-year period, followed by a decreasing trend afterward [32]. It has been demonstrated that age, gender, ortho-k lens experience and contact lens care habits are the potential risk factors for ortho-k-related keratitis [31]. Other treatments similar to the ortho-k lens also have side effects. Lin et al. reported that atropine-treated children may suffer photophobia and increasing IOP and that there is a risk of inducing crowding of the anterior chamber angle [22]. There were no absolute conclusions about whether the ortho-k lens or another treatment was better. However, we suggest that in terms of controlling myopia, the ortho-k lens is a useful method.

Certain limitations existed in the present study. First, the sample size of this study was relatively small. To confirm the potential efficacy and limitations of long-term ortho-k treatment, a larger-scale study must be conducted. Second, factors that may affect myopic progression, such as pupil size, accommodative lag, retinal image quality, peripheral refractive status, and a history of parental myopia, were not considered in our study. Further studies should focus on these factors. Third, the study period was not long because the data were from the medical records of children who came to our clinic for vision correction using spectacles or ortho-k lenses from 2013 to 2014. However, subsequent follow-up will still be performed for further study.

## Conclusion

In conclusion, the current study confirms that the ortho-k lens is a promising treatment method to control myopia in children with low to moderate myopia. In particular,this lens offers more benefits to younger children with fast progression and allows better control of higher degrees of myopia. Although the ortho-k lens cannot completely arrest axial elongation in myopic children, it can retard it to a certain extent, which suggests that this treatment is a good choice for myopic children for whom wearing the ortho-k lens is suitable.

## Abbreviations

BCVA, best-corrected visual acuity; IOL, intraocular lens; IOP, intraocular pressure; M/F ratio, male/female ratio; Non-RCTs, nonrandomized controlled trials; Ortho-k, orthokeratology; SER, spherical equivalent refractive error; VA, visual acuity
